# Meeting abstracts from the Annual Conference Clinical Genetics of Cancer 2018: Szczecin, Poland. 11-12 October 2018

**DOI:** 10.1186/s13053-018-0101-5

**Published:** 2019-02-08

## A1 Search for new genomic changes associated with high risk of cancer

### Cybulski C^1^, Kluźniak W^1^, Huzarski T^1^, Wokołorczyk D^1^, Rusak B^1^, Stempa K^1^, Kashyap A^1^, Jakubowska A^1^, Szwiec M^2^, Dębniak T^1^, Gronwald J^1^, Narod SA^3^, Akbari MR^3^, Lubiński J^1^, the Polish Hereditary Breast Cancer Consortium

#### ^1^Department of Genetics and Pathology, Pomeranian Medical University in Szczecin, Szczecin, Poland; ^2^Clinics of Oncology, University Hospital in Zielona Góra, Zielona Góra, Poland; ^3^Women’s College Research Institute, Toronto, ON, Canada

Hereditary cancer is estimated to account for up to 10% of the worldwide cancer burden and breast cancer is one of the top cancers with a large proportion (10-15%) of hereditary cases. Many cancer-predisposing genes are involved in the balance between cell growth and cell death or in maintaining genome integrity. There are about 500 known cancer-causing genes with reported mutations in somatic and/or germline DNA. Of those genes about 100 have been shown to be mutated in germline DNA of human and could be inherited from parents to their children and predispose them to hereditary cancers. Among genes which are associated with inherited cancers and BRCA1, BRCA2, CHEK2, PALB2 and NBS1. Identification of predisposing mutations enables specific management including prevention, early detection and treatment. Cancers that arise in mutation carriers usually have specific clinical characteristics, prognosis and sensitivity to treatment.

With the advent of deep sequencing of DNA and whole-exome sequencing, it is now possible to apply a powerful new technology to identify new mutations associated with a high risk of cancer. Now, we studied (grant funded by Narodowe Centrum Nauki, registration project number 2015/17/B/NZ5/02543) a large series of women with breast cancer from families with clustering of breast cancer (families with hereditary breast cancer – HBC) using TaqMan-PCR and multiplex-PCR (715 cases) and using whole exome sequencing (617 cases). This study led to identification of spectrum of breast cancer predisposing mutations in a large group of Polish families with HBC.

## A2 Cancer genetic testing and counselling on the move

### Sijmons R^1^

#### ^1^Department of Genetics, University of Groningen, University Medical Center Groningen, Groningen, The Netherlands

The field of familial cancer genetics is continuously on the move. In this presentation I will highlight three topics that are of growing interest. The first topic is the use of gene panels as a means not to only test for the genes that are related to the cancer that triggered referral, i.e. diagnostic testing, but also to screen all other genes on that panel for possible mutations. A recent development in the Netherlands is to perform whole genome sequencing of both tumour and lymphocytes in cancer patients; the latter with the goal to help selecting the somatic variants. However this germline WGS would offer the possibility to screen for a wide range of genetic conditions, relevant for patients and their families. There are many pros and cons to be considered. The second topic is the mainstreaming of genetic testing, i.e. DNA testing being ordered by non-geneticist clinicians, e.g. medical oncologists and surgeons. Several models are now being tested in the field in various countries, typically for breast cancer. The third topic is the use of polygenic risk scores for cancer, as opposed to the traditional testing of genes for Mendelian, single gene, disorders. Something that was for many years regarded as being a predominantly genetic-epidemiological scientific exercise, is now being adopted for clinical application.

## A3 A genetic variant in *telomerase reverse transcriptase (TERT)* modifies cancer risk in Lynch syndrome patients harbouring pathogenic *MSH2* mutations

### Talseth-Palmer BA^1,2^ , Evans TJ^1^, Belhadj S^3^, Bolton KA^1^, Dymerska D^4^, Changur SJ^5^, Kurzawski G^4^, Wijnen JT^5^, Valle L^3^, Vasen HFA^7^, Lubinski J^4^, Scott RJ^1, 8^

#### ^1^School of Biomedical Science and Pharmacy, Faculty of health and Medicine, University of Newcastle and Hunter Medical Research Institute, Newcastle, Australia; ^2^Research and Development Unit, Møre and Romsdal Hospital Trust, Molde, Norway; ^3^Hereditary Cancer Program, Catalan Institute of Oncology, IDIBELL and CIBERONC, Hospitalet de Llobregat, Barcelona, Spain; ^4^Hereditary Cancer Center, Pomeranian Medical University, Szczecin, Poland; ^5^Department of Human Genetics, Leiden University Medical Center, Leiden, the Netherlands; ^6^Department of Clinical Genetics, Leiden University Medical Center, Leiden, the Netherlands; ^7^Department of Gastroenterology and Hepatology, Leiden University Medical Center, Leiden, the Netherlands; ^8^NSW Health Pathology, Newcastle, NSW, Australia

Background

Individuals with Lynch syndrome (LS), an autosomal dominant inherited cancer syndrome caused by mutations in DNA mismatch repair genes have an increased risk of developing a range of epithelial malignancies, in the absence of a pre-malignant phenotype. Common genetic variants of the TERT gene are associated with telomere length and have been linked to a wide range of cancers, including colorectal cancer (CRC) and with tumours arising in LS.

Aim

In this study we have genotyped 3 SNPs in *TERT*; rs2736108 (upstream gene variant), rs2075786 and rs7705526 (both intronic variants), previously shown to influence telomere length in tumours and in association with LS.

Methods

We genotyped 1895 LS patient samples for rs2075786 (G>A) and 1241 LS patient samples for rs2736108 (C>T) and rs7705526 (C>A) using TaqMan SNP assays (Applied Biosystems). The risk of cancer with each SNPs’ genotype was estimated by heterozygous and homozygous odds ratio (OR) using simple logistic regression and mixed-effects logistic regression to adjust for gene, gender and country taking into account family ID (probands and relatives).

Results

We observed an increased risk of cancer in patients carrying pathogenic *MSH2* mutations and the heterozygous genotype (GA) for rs2075786 (OR=1.84, confidence interval (CI) =1.15-2.94), p=0.01). This association is even stronger if patients <45 years of age at diagnosis were compared to cancer free patients; *MSH2* and GA for rs2075786 (OR=2.53, CI=1.43-4.49, p=0.002).

Conclusion

Even though both *MLH1* and *MSH2* mutation carrier’s initially have similar risks of cancer, a SNP in *TERT* appears to be associated with a differential risk of developing cancer for *MSH2* mutation carriers. *MSH2* deficiency alone has previously been shown to accelerate telomere shortening in normal human cells and can explain the increased risk in younger heterozygous *MSH2* mutation carriers. By including modifier gene/loci in risk algorithms it should be possible to tailor surveillance options for individual patients.

## A4 Mutations of genes predisposing to occurrence of intestinal polyposis in Poland

### Pławski A^1,2^, Lis E^1^

#### ^1^Institute of Human Genetics, Polish Academy of Sciences, Poznań, Poland; ^2^Department of General, Endocrinological Surgery and Gastroenterological Oncology, Poznan University of Medical Sciences, Poznań, Poland

The term polyp refers to any tissue hypertrophy from the surface of mucous membranes. Intestinal polyps arise from the mucous membrane of the small and large intestines. The hyperplastic, adenomatous, hamartomatous and inflammatory polyps are main types of polyps observed in gastrointestinal tract. The adenomatous and hamartomatous polyps may occur as symptoms of susceptibility syndromes to the occurrence of neoplastic diseases. The syndromes of inherited predispositions associated with the presence of multiple intestinal polyps include: familial adenomatous polyposis (MIM 175100), Peutz-Jeghers syndrome (MIM 175200), juvenile polyposis syndrome (MIM 174900) and Cowden Syndrome(MIM 153480). The occurrence of these diseases is associated with mutations of the following genes: APC, MUTYH, STK11, BMPR1A, SMAD4 and PTEN. The spectrum of point mutations and copy number variation in genes predisposing to intestinal polyposis in the Polish population were determined. Determination of the spectrum of mutations of predisposition genes in the Polish population allows to optimize mutation detection for the Polish population. The research in part was financed by the project NCN 2013/09 / N / NZ5 / 02505.

## A5 Association between mutations in genes from NGS multi-gene panels and breast and ovarian cancer risk

### Suszynska M^1^, Klonowska K^1#^, Kozlowski P^1^

#### ^1^Department of Molecular Genetics, Institute of Bioorganic Chemistry, Polish Academy of Sciences, Poznan, Poland; ^#^Present address: Brigham and Women's Hospital, Harvard Medical School, Boston, Massachusetts, USA

Background

As *BRCA1/2* mutations are responsible for only part of familial breast cancer (BC) and ovarian cancer (OC) cases, researchers and clinicians are looking for other BC/OC risk genes. Recent progress and decreasing cost of the next generation sequencing (NGS) has allowed to expand the range of examined genes. However, the inclusion of additional genes to the NGS multi-gene panels (MGPs), is not always supported by strong genetic or statistical evidences and most of the genes still have to be considered as “candidates”.

Aim of the study

We aimed to estimate a reliable BC and OC risk associated with mutations in genes repeatedly employed in MGPs.

Methods

We accomplished a wide-scale meta-analysis of results from 48 MGP-based studies, that analyzed BC and OC patients. The mutation frequency of ~120,000 BC/OC cases and ~120,000 controls, extracted from public gnomAD database, were used to estimate BC and OC association with mutations in 37 genes.

Results

In total, 13 and 11 of the analyzed genes were significantly associated with an increased BC and OC risk, respectively. We noticed that mutations in a few genes are attributed to a high BC risk, at a level similar to that of *BRCA2* mutations. Furthermore, our results revealed that *CDKN2A*, not often mentioned in the context of BC, can be classified as a high-risk gene. The other striking observation of our study is the substantial difference in the profile of genes contributing to either BC or OC risk. We showed that mutations in several genes much more strongly predispose to OC than BC. Extreme examples are mutations in *RAD51D*, *RAD51C*, and *NBN* that are specific to OC and do not predispose to BC at all. Additionally, what is equally important, the analysis indicated which genes, frequently used in MGPs, are not associated with BC/OC risk.

Conclusions

In summary, our results define with high confidence the role of several genes in the genetic predisposition to BC and OC. The practical implication of our results is the support that they provide for a substantively justified interpretation of diagnostic results.

Acknowledgments: This work was supported by grants from the Polish National Science Centre (NCN 2015/17/B/NZ2/01182 awarded to Piotr Kozlowski).

## A6 Constitutional methylation of *BRCA1* gene and breast cancer risk

### Prajzendanc K^1^, Domagała P^2^, Ryś J^3^, Huzarski T^1^, Szwiec M^4,5^, Tomiczek-Szwiec J^5,6^, Redelbach W^5,7^, Sejda A^8^, Gronwald J^1^, Fudali L^9^, Wiśniowski R^10^, Hybiak J^2^, Łukomska A^1^, Białkowska K^1^, Sukiennicki G^1^, Wojdacz TK^11^, Lubiński J^1,12^, Jakubowska A^1,13^

#### ^1^Department of Genetics and Pathology, International Hereditary Cancer Center, Pomeranian Medical University, Szczecin, Poland; ^2^Department of Pathology, Pomeranian Medical University, Szczecin, Poland; ^3^Department of Tumor Pathology, Maria Skłodowska-Curie Memorial Centre and Institute of Oncology, Cracow, Poland ; ^4^Department of Clinical Oncology, University Hospital in Zielona Gora, Poland; ^5^Faculty of Natural Sciences and Technology University of Opole, Opole, Poland; ^6^Department of Oncological Gynecology, Oncology Center in Opole, Opole, Poland; ^7^Oncology Surgery Department, Oncology Center in Opole, Opole, Poland; ^8^Department of Pathology, Provincial Specialist Hospital, Olsztyn, Poland ; ^9^Clinical Department of Pathology, Frederick Chopin Clinical Provincial Hospital No 1, Rzeszow, Poland; ^10^Department of Clinical Oncology, Regional Oncology Center, Bielsko-Biala, Poland; ^11^Aarhus Institute of Advanced Studies, University of Aarhus, Aarhus, Denmark; ^12^Read-Gene S.A., Grzepnica, Polska; ^13^Independent Laboratory of Molecular Biology and Genetic Diagnostics, Pomeranian Medical University, Szczecin, Poland

Methylation of CpG islands in promoter region of genes is an epigenetic modification that causes silencing of genes and might be associated with cancer risk if it is present in peripheral blood. It has been suggested that constitutional methylation of *BRCA1* promoter correlates with breast cancer risk, especially with triple-negative tumors.

In this study we evaluated an association of *BRCA1* methylation in peripheral blood with breast cancer risk and assessed correlation with clinical features of tumors. We examined three groups of women: 504 triple-negative breast cancer cases, 438 non-TNBC cases and 500 healthy controls. All women were negative for 13 common Polish *BRCA1* germline mutations. Moreover, 274 FFPE tumor tissues were tested to estimate association between constitutional and somatic *BRCA1* promoter methylation. Methylation status was assessed using methylation-sensitive high-resolution melting (MS-HRM). Additionally, we genotyped variant c.-107A>T in *BRCA1* gene to assess its potential correlation with *BRCA1* methylation.

The results show that *BRCA1* methylation detected in peripheral blood is significantly associated with the risk of TNBC (OR 5.25, p<0.001) and correlates with methylation in paired tumors. The variant c.-107A>T was not detected in tested women from Polish population.

The study was supported by the National Science Centre (NCN) grant 2014/15/B/NZ1/03386.

## A7 *BRCA1/2, CHEK2, PALB2* and *RAD51C* mutations in ovarian cancer patients from Polish population

### Łukomska A^1^, Peruga N^1^, Białkowska K^1^, Prajzendanc K^1^, Sukiennicki G^1^, Kluźniak W^1^, Menkiszak J^2^, Jasiówka M^3^, Gronwald J^1^, Huzarski T^1^, Tomiczek-Szwiec J^4,5^, Szwiec M^4,6^, Cybulski C^1^, Lubiński J^1,8^, Jakubowska A^1,7^

#### ^1^Department of Genetics and Pathology, International Hereditary Cancer Center, Pomeranian Medical University, Szczecin, Poland; ^2^Department of Surgical Gynecology and Gynecological Oncology of Adults and Adolescents, Pomeranian Medical University, Szczecin, Poland; ^3^Department in Cracow, Oncology Cancer Center Institute Marie Skłodowska-Curie, Cracow, Poland; ^4^Faculty of Natural Sciences and Technology University of Opole, Opole, Poland; ^5^Department of Oncological Gynecology, Oncology Center in Opole, Opole, Poland; ^6^Department of Clinical Oncology, University Hospital in Zielona Gora, Poland; ^7^Independent Laboratory of Molecular Biology and Genetic Diagnostics, Pomeranian Medical University, Szczecin, Poland; ^8^Read-Gene SA, Grzepnica, Poland

In Poland ovarian cancer represents the second cause of cancer among gynecological malignancies and the fourth cause of cancer deaths among women. Literature data shows that more than one-fifth of ovarian cancer cases have been related to hereditary factors. It has been recognized that the most frequently germline mutation in hereditary ovarian cancer are BRCA1/2 mutations. Nevertheless, several other genes, as RAD51C, have been suggested to be associated with hereditary ovarian cancer. Mutations in other genes which are known to be associated with high breast cancer risk in Polish population, as PALB2 and CHEK2, have not been tested in ovarian cancer patients up to now.

The aim of the study is to estimate the frequency of recurrent Polish germline mutations in BRCA1/2, RAD51C, PALB2 and CHEK2 genes among unselected and familial ovarian cancer patients. Additionally, an association of RAD51C, PALB2 and CHEK2 mutations with ovarian cancer risk was assessed.

Molecular analyses included genotyping of recurrent mutations in BRCA1/2 (13), RAD51C (3), PALB2 (2) and CHEK2 (3) in a group of ~2000 unselected OC, ~250 HOC and 2000 healthy controls .

The frequency of BRCA1/2 was 11.02%. We found significant association of RAD51C and PALB2 mutations, but not CHEK2 mutations, with ovarian cancer risk.

The study was supported by the „Młody Badacz” grant MB-158-219/17

## A8 The presence of *NOD2* mutation in younger breast cancer patients – single center experiences

### Huszno J^1,2^, Kołosza Z^3^, Lisik M^1^, Nycz-Bochenek M^1^, Grzybowska E^4^

#### ^1^Genetic Outpationt Clinic, Maria Sklodowska-Curie Memorial Cancer Center and Institute of Oncology, Gliwice Branch, Gliwice, Polan; ^2^I Radiation and Clinic Oncology Department, Maria Sklodowska-Curie Memorial Cancer Center and Institute of Oncology, Gliwice, Gliwice, Poland; ^3^Department of Medical Physics, Maria Sklodowska-Curie Memorial Cancer Center and Institute of Oncology, Gliwice Branch, Gliwice, Poland; ^4^Center for Translational Research and Molecular Biology of Cancer, Maria Sklodowska-Curie Memorial Cancer Center and Institute of Oncology, Gliwice Branch, Gliwice, Poland

Introduction

The population risk of breast cancer before the age of 50, associated with the *NOD2* mutation, is approximately 1%. It increases 5 times the risk of DCIS <50 years. The purpose of this study was to evaluate the presence of *NOD2* (c.3016_3017insC) mutation in younger breast cancer (BC) patients (<45 years) according to clinicopathological factors in comparison to control group.

Material

We have were analyzed prognostic factors in younger BC patients with confirmed *NOD2* (c.3016_3017insC) (n=42) mutation. Control group was selected from BC patients without tested mutations (n=392). The presence of the most common mutations in *BRCA1* (c.68_69delAG, c.181T>G, c.4034delA, c.5266dupC, c.3700_3704del5), *BRCA2* (c.5946delT and c.9403delC), CHEK2*1100delC or I157T mutations genes were excluded. Mutation analysis was carried by a multiplex allele-specific polymerase chain reaction assay.

Results

The median age at breast cancer diagnosis for the carriers of the *NOD2* mutation was 47 years (from 27 to 68) and for control group 53 years (from 26 to 78). 51 patients were in age under 45 years 16 (38%) in *NOD2* mutation carriers and 42 (22%) in control group, p=0,040. Gastrointestinal cancers in family history (31% vs. 7%, p=0.012), especially colorectal cancer (13% vs. 1%, p=0.061) were reported more frequently in patients with *NOD2* mutations. There was also observed tendency to the presence of breast cancer in family history in *NOD2* mutation carriers (19% vs. 9%, p=0.369) (age <45 years). In group of patients in age >45 years cancers in family history, including breast cancer were observed more often in *NOD2* mutation carriers in comparison to control group of patients (73% vs. 31%, p=0.0001, for breast cancer in family history: 42% vs. 10%, p=0.0001).

HER2 overexpression was reported significantly more often in control group (51.1% vs. 6.3%; p=0.007). In contrary, there was no differences between *NOD2* mutation carriers and control group according to ER (31% vs. 40%, p=0.587) and PR (38% vs. 43%, p=0.787) negative steroid receptor status. Breast cancer subtype A was more frequent in *NOD* carriers compared to control group (38% vs. 7%, p = 0.003). Lower histological grade G1-G2 (56% vs. 68%, p=0.395) and lymph nodes without metastases (69% vs. 48%, p=0.174) were observed similarly frequently in *NOD2* mutation carriers than control group.

Conclusion

The presence of *NOD2* mutations (in age <45 years) were associated with younger age of disease diagnosis and gastrointestinal cancer in family history. Breast cancer in family history was characteristic for *NOD2* mutation carriers in age>45 years. Luminal A BC subtype was most characteristic for this group.

## A9 Clinical and molecular aspects of hereditary breast cancer diagnosis and management: PALB2 and RECQL epidemiology in Latvia, Manchester scoring system and contralateral breast cancer risk reduction

### Hilz P^1^ , Heinrihsone R^1^, Pätzold LA^1^, Qi Q^2^, Trofimovics G^3^, Gailite L^4^, Peteris Loza^1^, Elina Tauvena^1^, Arvids Irmejs^1^, Janis Gardovskis^1,3^, Edvins Miklasevics^1^, Zanda Daneberga^1^

#### ^1^Institute of Oncology, Riga Stradins University, Riga, Latvia; ^2^Department of Integrative Oncology,Fudan University Shanghai Cancer Center, Shanghai, China; ^3^Department of Surgery, Riga Stradins University, Riga, Latvia; ^4^Scientific Laboratory of Molecular Genetics, Riga Stradins University, Riga, Latvia

Here we are going to present three small studies covering different aspects of hereditary breast cancer management.

Large-scale case control studies revealed a number of moderate risk - low frequency breast cancer alleles of the *PALB2* and *RECQL* genes. Some of them reported as founder variants of Central and Eastern Europe. Based on highly similar founder variant spectra of the *BRCA1* in Poland and Latvia, we decided to test frequency of other common variants of moderate breast cancer risk - c.509_510delGA (rs515726124) and c.172_175delTTGT (rs180177143) of the *PALB2* gene and c.1667_1667+3delAGTA variant of the *RECQL* gene in breast cancer case-control series from Latvia to gain better understanding of the role of genes in susceptibility to breast cancer and their clinical significance. The calculated frequency for c.509_510delGA of the *PALB2* gene in the case group is 0.35% and 0.00% in the control group, with respective relative risk (RR) 7.18 (CI 95% 0.37 – 138.75; *p* = 0.19). As for *PALB2* c.172_175delTTGT variant, the frequency in the case group of our study is 0.04%. In the control group of our study non heterozygous carriers were detected, which lead to calculated RR = 1.50 (CI 95% 0.06 – 36.83; *p*-value = 0.80). There were no carriers of the *RECQL* variant c.1667_1667+3delAGTA identified in our case group and 2 heterozygotes were identified in the control group. The calculated RR = 0.26 (CI 95% 0.01 – 5.33; *p*-value = 0.38).Acquired results on the *PALB2* gene variants are able to supplement evidence on the allele frequency in the breast cancer patients. Based on our results we cannot confirm contribution of the *RECQL* variant c.1667_1667+3delAGTA allele to the breast cancer development.

Recent availability of commercial complete BRCA1/2 testing in BRCA1 founder (c.4035delA, c.5266dupC) population like Latvia and relatively high non-founder BRCA1/2 mutations frequency has raised question about the selection criteria for this service, if BRCA1 founder mutations are negative. Wide variety of reasons contributes to low diagnostic accuracy of family cancer history criteria alone in our population. Aim of the study is to evaluate the diagnostic value of Manchester scoring system (MSS). MSS was calculated in 1006 unselected breast cancer cases. 57/1006 (5.7%) MSS positive cases were identified. 24/57 MSS positive cases were BRCA1 founder positive, but 33/57 – negative. From the other hand there are 36/1006 (3.6%) BRCA1 founder mutation carriers in our cohort. 24/36 has positive MSS and 12/36 negative MSS. Our conclusion is that MSS has higher diagnostic accuracy in comparison to family cancer history alone and 3.3% of unselected MSS positive and BRCA1 founder mutation negative breast cancer cases should undergo complete BRCA1/2 testing as probability of finding pathogenic mutation is more than 10%.

BRCA1 positive breast cancer cases has more frequently contralateral breast cancer (CBC) events. The most effective prevention strategy is contralateral risk reductive mastectomy (CRRM). Since 2009 22 CRRMs have been performed in BRCA1 positive unilateral breast cancer cases. Control group consist of 21 BRCA1 positive unilateral breast cancer case without CRRM. Median follow-up since treatment of primary breast cancer is 4.29 +/- 0.78 years. There are 5/21 (24%) cases of CBC in the control group, but no CBC events 0/22 (0%) in CRRM group. Our conclusion is that BRCA1 positive breast cancer patients have high frequency of CBC events and CRRM is effective method to reduce CBC events in BRCA1 positive breast cancer cases.

## A10 *PALB 2* mutation in a woman with breast cancer: a case report

### Lisik M^1^, Tęcza K^1^, Pamuła-Piłat J^1^, Kalinowska-Herok M^1^, Mazur M^1^, Łanuszewska J^1^, Grzybowska E^1^

#### ^1^Maria Skłodowska-Curie Memorial Cancer Center and Institute of Oncology Gliwice Branch, Gliwice, Poland

Partner and localizer of BRCA 2 (*PALB2*) was identified as a moderate-risk gene of breast and pancreas cancer .*PALB2* mutations are rare. The prevalence of *PALB2* mutation is now estimated to be 1,7 % in Southern Poland. The absolute risk of breast cancer in women up to 70 years of age with *PALB2* mutation ranges from 33% for women without family histories of breast cancer to 58 % for women with family history. In the present study, one patient with a deleterious mutation of *PALB2* (c.509-510delGA) has been identified. The proband was a 47-years old woman was admitted to Institute of Oncology because of enlargement of right breast. Contrast-enhanced MRI of the right breast showed a large high dense soft mass lesion in the outer quadrant and center of the right breast with speculated outline measured (10 x 7,5 cm). Moreover, multiple enlarged axillary lymph nodes were visible. Pathological findings were as follows: invasive ductal carcinoma (cribriform type), histological grade 3,. an estrogen receptor-positive (ER-positive), progesterone receptor-positive (PgR-positive), and human epidermal growth factor receptor 2-positive (HER2-positive) and an estrogen receptor-negative (ER-negative), progesterone receptor-negative (PgR-negative), and human epidermal growth factor receptor 2-positive (HER2-positive). The proband had mother who was diagnosed with colon cancer at the age of 65 and maternal brother who was diagnosed with gastric cancer at the age of 55 years.

The patient gave written informed consent for the publication of her clinical data.

## A11 Brca1-related multiple primary cancers in Belarus

### Savanevich AL^1^, Stepuro TL^1^, Shulga AV^1^

#### ^1^Grodno State Medical University, Grodno, Republic of Belarus

The study of primary-multiple tumors allows us to come closer to understanding both the differences existing between individual tumors, and their similarity, based on the unity of a significant number of risk factors. The aim of the study was to study the clinical and diagnostic features of primary-multiple tumors with ovarian involvement. In 81 patients included in the study, 174 malignant tumors were diagnosed, one of which was localized in the ovaries. Primary multiplicity of neoplasms in most cases was represented by two localizations - 70 cases (86%), less often three - 10 patients (12%). Often the development of ovarian cancer was combined with breast cancer - 27 cases (33%), uterine malignant tumors - 23 (28%) and gastrointestinal tract tumors - 16 (20%). The study of family history indicates the presence of a hereditary predisposition to the development of tumors in 36 women (44%). Only in 16% of cases relatives of the first line had ovarian and / or breast cancer. Twenty BRCA1 germ-line mutation carriers (25%) were identified by the analysis of the Slavic founder alleles in BRCA1 gene. Among these were the most frequently detected mutations 5382insC (55%) and 4153delA (25%). Among women with breast and ovarian cancer, the mutation in the BRCA1 gene was confirmed in 56% of cases, which confirms the importance of genetic factors in the development of primary-multiple tumors of the female reproductive system. In this work, the characteristics of synchronous and metachronous primary-multiple tumors are presented. When studying the receptor status of BRCA1-associated primary-multiple tumors of the ovaries and mammary gland, it was found that most ovarian carcinomas, unlike breast tumors, have a receptor-positive status. A burdened family history and the identification of mutations in the BRCA1 gene should be considered as an integral part of a comprehensive survey of women with malignant neoplasm of the female reproductive system to determine the genetic risk of developing new tumors of the female reproductive system and develop the principles of genetic cancer prevention. Features of the receptor status of BRCA1-related ovarian carcinomas allow each patient to be treated differently, taking into account the genetic and receptor status of a specific tumor.

## A12 CHEK2 c.444+1G>A variant and papillary thyroid cancer – own study and meta-analysis

### Kula D^1^, Kalemba M^1^, Puch Z^1^, Kowalska M^1^, Jarząb M^2^, Sznajder D^1^, Oczko-Wojciechowska M^1^, Rusinek D^1^, Żebracka-Gala J^1^, Halczok M^1^, Tyszkiewicz T^1^, Pawlaczek A^1^, Cyplińska R^1^, Jarząb B^1^

#### ^1^Department of Nuclear Medicine and Endocrine Oncology, Maria Sklodowska-Curie Institute, Oncology Center, Gliwice Branch, Gliwice, Poland; ^2^3rd Department of Radiotherapy and Chemotherapy, Maria Sklodowska-Curie Institute - Oncology Center, Gliwice Branch, Gliwice, Poland.

Background

Genetic predisposition to papillary thyroid cancer (PTC) is known to be muligenetic and complex with many genes interacting with environmental factor. However, the genes responsible for this predisposition mostly are not known. Many of them were analysed, but only for some genes the association with thyroid cancer is well established. Among analysed genes was CHEK2, but with the relatively small number of 468 PTC cases included. The aim of our study was to analyse the association of c.444+1G>A (formely IVS2+1G>A) CHEK2 variant in the big number of 2279 of PTC cases and 1218 controls. c.444+1G>A variant was analysed with HRM methods and confirmed by Sanger sequencing. The second purpose of the study was to perform the meta-analysis in all available Polish data to summarize c.444+1G>A CHEK2 variant association with PTC.

Results

A significant association was seen for c.444+1G>A with OR=4.49. Performed meta-analysis have confirmed these results - for c.444+1G>A association was seen with OR=5.89.

Conclusions

We have confirmed the association of c.444+1G>A CHEK2 variants with PTC in Polish population.

This work was supported by the National Centre for Research and Development project under the program “Prevention practices and treatment of civilization diseases” STRATEGMED (STRATEGMED2/267398 /4/NCBR/2015) and National Science Center, Polish, grant number N N402 193740

## A13 Evaluation of the effectiveness of recruitment methods for prophylactic examinations in groups of high-risk cancer patients

### Galor A^1^, Cezary C, Lubiński J1, Narod SA^2^, Gronwald J^1^

#### ^1^Department of Genetics and Pathology, International Hereditary Cancer Center, Pomeranian Medical University, Szczecin, Poland; ^2^Women’s College Research Institute, Toronto, ON, Canada

Introduction

Literature data indicate that the use of appropriate recruitment methods for prevention programs has an impact on the recruitment and cost effectiveness of these programs. In Poland, the effectiveness of active recruitment methods for preventive screening programs in high-risk cancer groups, which would include gene mutation carriers, has not been evaluated so far

Objective

The aim of the study was to assess the telephone, postal and combined (letter invitation and subsequent telephone invitation in the absence of responses to a letter invitation) method of invitations to preventive programs in groups of patients at high risk of developing malignant tumors, including recruitment and cost effectiveness.

Material and methods

The research was carried out in the following groups of patients:

Group I, 93 males with a BRCA1 gene mutation; Group II, 199 women with a BRCA1 gene mutation; Group III, 1497 women with the pedigree diagnosis of HBC or HBO; Group IV, 419 women with the pedigree diagnosis of HNPCC or LOFCC.In each of the examined groups (Group I-IV), participation in preventive examinations (recruitment effectiveness) depending on the form of the invitation was determined and the economic effectiveness of the patient invitation methods was evaluated.

Results

1. The telephone invitation is characterized by the highest recruitment and economic efficiency in all the examined groups (Group I-IV) in comparison to other methods of invitations.

2. Invitation by the letter method is characterized by the lowest recruitment effectiveness and indirect economic effectiveness in all examined groups (Group I-IV).

3. Invitation by the combined method is characterized by similar recruitment effectiveness to the telephone method and is associated with the highest costs incurred in all examined groups (Group I-IV).

4. In the group of women, who carry BRCA1 gene mutation, the highest economic efficiency was observed using all the tested methods and the highest recruitment efficiencyusing the telephone and letter method.

5. The lowest recruitment effectiveness is observed in the group of patients with pedigree diagnosis HNPCC or LOFCC implemented through a letter invitation.

6. The highest recruitment costs are observed in the group of males, carriers of the BRCA1 gene mutation using the combined method.

Conclusions

1. The type of invitations method used affects the effectiveness of recruitment for preventive examinations.

2. The type of invitations method used has an impact on the cost effectiveness of recruitment for preventive examinations.

3. Telephone methods should be a recommended way of inviting patients from high-risk cancer groups for prophylactic examinations.

4. In groups of high-risk cancer patients, it is possible to increase economic efficiency and probably recruitment by introducing new methods of invitations using correspondence via email (e-mail) and short message service via mobile telephony (SMS). In this case, the combined method can potentially have an advantage over other methods. Further studies on the effectiveness of these methods are necessary.

## A14 Long-term survival of invasive ovarian cancer associated with BRCA1- 4153delA mutation in Lithuanian population

### Elsakov P^1^, Razumas T^3^, Luksyte A^2^, Smailyte G^2^

#### ^1^Institute of Oncology, Vilnius University, Vilnius, Lithuania; ^2^Department of Breast Surgery and Oncology, Institute of Oncology, Vilnius University, Vilnius, Lithuania; ^3^Vilnius Maternity Hospital, Santariškiu g. 2, LT-08661 Vilnius, Lithuania.

The aim of this study was to estimate 10-year survival for women with invasive ovarian cancer associated with BRCA1-4153delA mutation in a high incidence (Lithuanian) population.

Materials and methods

The study focused on 71 ovarian cancer patients treated at Vilnius University Oncology Institute. Sixty patients (Group I) were consecutive, newly diagnosed cases, unselected for age or family history. Eleven patients (Group II) were selected by strong hereditary criteria with an aggregation of at least three breast/ovarian cancers had Hereditary Breast Ovarian Cancer Syndrome (HBOCS). The founder mutations of BRCA1 carrier status and BRCA2 of these patients were identified. The treatment of all patients was similar as per national protocols for treatment of ovarian cancer cases independent of mutation status. Only a patients alive 10 years after diagnosis were included in the study. Kaplan–Meier survival curves were constructed for the groups: Group I – unselected women with ovarian cancer and Group II – women with HBOCS by heredity and BRCA1/2 mutation status. The Log-Rank test was used to evaluate the statistical significance of differences. A p < 0.05 was indicative of a significant statistical difference. Cox-proportional Hazards models were used to estimate Hazard Ratios (HRs) associated with mutation status.

Results

Overall survival 10-years after diagnosis showed no difference (p=0.4351) with regards to the presence or absence of a BRCA1/2 mutation for patients with invasive ovarian cancer. Ten year survival of those associated with BRCA1-4153delA mutation was similar (p=0.8918) to hereditary cases. Multivariable survival analysis for histologic subtype (serous and other) was also assosiated with a similar prognosis (p=0.579) at 10 years for hereditary and non-hereditary cases [HR=1.34, 95% CI= 0.47 to 3.80].

Conclusion

The results of our study show that the long-term (10 year) survival of patients with invasive ovarian cancer with BRCA 1/2 mutation was associated with a similar (p=0.4351) prognosis to those who were not curriers. The 10-year survival of the HBOCS case patients was similar (p=0.8918) to those associated with BRCA1-4153delA mutation when identical management and treatment were received.

## A15 Serum and blood trace metal levels as prognostic marker of survival in laryngeal cancer

### Lubiński Jakub^1^, Jaworowska E^1^, Derkacz R^2^, Marciniak W^2^, Lubiński Jan^2,3^

#### ^1^Clinic of Otolaryngology, Pomeranian Medical University, Szczecin, Poland; ^2^ReadGene, Grzepnica, Poland; ^3^Department of Genetics and Pathology, International Hereditary Cancer Centre, Pomeranian, Medical University, Szczecin, Poland

Introduction

There is a lot of literature data showing that metals can affect the development of cancer, including laryngeal cancer. However, their blood / serum levels have not yet been evaluated as a prognostic marker in laryngeal cancer. The aim of the study is a prospective evaluation of the correlation between results of treatment of patients with laryngeal cancer and levels of metals in the blood and serum.

Material and methods

Study groups: 1) 315 patients treated surgically in the period from July 2009 to February 2017 due to squamous cell carcinoma of the larynx, from whom blood was collected before the beginning of treatment in order to assess the levels of zinc, iron and copper in the serum.

2) 184 patients treated surgically in the period from January 2012 to February 2017 due to squamous cell laryngeal cancer, from whom blood was collected before the beginning of treatment to assess the levels of zinc, iron, copper, arsenic, cadmium, mercury and lead in the whole blood .

Clinical information on the age of onset, sex, clinical stage, radiation therapy, chemotherapy and pack-years was collected from all patients.

To determine the levels of the indicated metals, the technique of inductively coupled mass spectroscopy (ICP-MS) was used.

The results of the treatment were evaluated on the basis of the number of deaths that occurred during the prospective observation period.

The test groups were divided into three parallel subgroups (tertiles) depending on the levels of individual metals. The relationship between blood / serum metal levels and survival was analyzed statistically uni and multivariate, taking into account the influence of age, sex, clinical stage, chemotherapy, radiotherapy and pack-years.

Results

1. Zinc level in the serum: statistically significant increased risk of death in patients with the lowest zinc levels (<581 μg /l) in comparison with patients with the highest levels (> 688 μg /l): OR-2.04; p-0.029; HR-2.02; p <0.01.

2. Zinc level in the blood: statistically significant increased risk of death in patients with the lowest zinc levels (<5712 μg /l) in comparison with patients with medium levels (5716-6515 μg /l): OR- 3.15; p-0.01; HR-2.58; p <0.01.

3. Cadmium level in the blood: statistically significant increased risk of death in patients with mean cadmium levels (0.84-1.3 μg /l) in comparison with patients with the lowest levels (<83 μg /l): OR-2, 81; p-0.039; HR-2.14; p-0.043.

4. There were no statistically significant differences between the patients' survival and the levels of copper and iron in the serum as well as copper, arsenic, lead and mercury in the blood.

Conclusions

The levels of zinc below 580 μg /l in the serum and below 5700 μg /l in the blood and the level of cadmium above 0.80 μg /l in the blood are associated with an increased risk of death of a patient with laryngeal cancer in Poland. The implementation of chemoprevention modifying the levels of the above-mentioned metals might improve the results of treatment of laryngeal cancer.

## A16 Does the selenium level affect overall survival in lung cancer?

### Pietrzak S^1^, Wójcik J^2^, Kashyap A^1^, Grodzki A^2^, Baszuk P^1^, Bielewicz M^2^, Marciniak W^3^, Wójcik N^2^, Dębniak T^1^, Pieróg J^2^, Gronwald J^1^, Kubisa B^2^, Sukiennicki G^1^, Deptuła J^1^, Waloszczyk P^4^, Jakubowska A^1^, Lener M^1^, Lubiński J^1,3^

#### ^1^Department of Genetics and Pathology, International Hereditary Cancer Center, Pomeranian Medical University, Szczecin, Poland; ^2^Department of Thoracic Surgery and Transplantation, Pomeranian Medical University, Szczecin, Poland; ^3^ReadGene, Grzepnica, Poland; ^4^Independent Laboratory of Pathology, Zdunomed, Szczecin, Poland

Purpose

Although the results of studies in populations with low selenium status indicate an inverse correlation between the selenium concentration in the body and the risk of the lung cancer, the effect of this microelement on survival rate with this disease has not been studied.

Material and methods

We conducted a prospective study of 302 patients diagnosed with lung cancer in Szczecin, Poland. Serum selenium was measured at the time of diagnosis, prior to treatment. Patients were followed from the date of diagnosis until death or up to 80 months.

Vital status was obtained by linkage to the Polish National Death Registry.

Results

In the Cox proportional hazards analysis performed for all individuals with lung cancer, the hazard ratio (HR) for death from all causes was 1.25 (95% CI 0.86 to 1.83, P=0.99) for patients in the lowest tertile of serum selenium, compared to those in the highest tertile. Among the patients with stage I of the lung cancer this relationship was significant (HR-2.73; P = 0.01) for selenium level in the lowest tertile (<57 μg/L) compared to tertile 3 (>69 μg/L, reference). The 80 months crude survival after diagnosis was 79.5% (95% CI: 68.5 – 92.4%) for individuals in the highest tertile and was 58.1% (95% CI: 45.1 - 74.9%) for individuals in the lowest tertile with stage I of lung cancer.

Conclusion

This study suggests that in patients undergoing treatment with stage I of lung cancer, serum selenium level (>69 μg/L ) may be associated with improved survival.

## A17 Zinc as a marker of cancer risk

### Białkowska K^1^, Marciniak W^2^, Derkacz R^2^, Muszyńska M^2^, Sukiennicki G^1^, Baszuk P^1,2^, Lener M^1^, Łukomska A^1^, Prajzendanc K^1^, Huzarski T^1^, Gronwald J^1^, Oszurek O^1^, Cybulski C^1^, Dębniak T^1^, Morawski A^2^, Jakubowska A^1,3^, Lubiński J^1,2^

#### ^1^Department of Genetics and Pathology, International Hereditary Cancer Center, Pomeranian Medical University in Szczecin, Szczecin, Poland; ^2^Read – Gene SA, Grzepnica, Poland; ^3^Independent Laboratory of Molecular Biology and Genetic Diagnostics, Pomeranian Medical University in Szczecin, Szczecin, Poland

Background

Zinc is a micronutrient, which is essential for human health, involved in regulation of gene expression, e.g. genes related to cell cycle, apoptosis, response to DNA damage, antioxidant defense, immune response. Literature data on zinc association with cancer risk show inconclusive results.

Aim of the study

The aim of the study was to evaluate the relationship between zinc blood levels and subsequent cancer risk in a large prospective cohort of persons followed for incident cases of cancer in Szczecin Poland.

Material and methods

The study was conducted in 3 prospective cohort consisted of persons with no cancer at that baseline:

1. 601 women with BRCA1 mutation, among them 42 cancers were identified during the follow-up
2. 1698 women without BRCA1 mutation, among them 110 cancers were identified during the follow-up
3. 1467 men, among them 42 cancers were identified during the follow-up.

Zinc level blood was measured by inductively coupled plasma mass spectrometry (ICP-MS) using Elan DRC-e ICP-Mass Spectrometer, Perkin Elmer. Odds Ratios were calculated using Fisher’s exact test.

Results

It was observed that female BRCA1 mutation carriers with Zn blood level 6300 – 6700 μg/L have 4-fold lower risk than carriers with Zn level <6300 μg/L or >6700 μg/L (regardless to age) (OR=0,24; 95%CI 0,06 – 1; p=0,03). Results from the cohort of women without BRCA1 mutation and cohort of men revealed that Zn blood level at which cancer risk is decreased is strongly dependent on age. Women <60 y/o with Zn blood level >6800 μg/L have 3-fold lower risk than women with Zn level <6800 μg/L (OR=0,3; 95%CI 0,09 – 0,97; p=0,04). For women >60 y/o a tendency to 3-fold reduced risk is observed with Zn blood level 5600 – 6000 μg/L comparing to Zn level <5600 or >6000 μg/L (OR=0,3; 95%CI 0,1 – 1,1; p=0,06). In the cohort of men individuals <60 y/o and Zn blood level 6300 - 6800 μg/L have 3-fold lower risk of cancer than men with Zn level <6300 μg/L or > 6800 μg/L (OR=0,31; 95% CI 0,11 – 0,89; p=0,02). Men >60 y/o and Zn blood level 7000 – 7300 μg/L have 10-fold lower risk of cancer than men with Zn level <7000 μg/L or >7300 μg/L (OR=0,1; 95% CI 0,01 – 0,72; p=0,003).

Conclusions

Results from this study suggest that blood zinc level is a strong marker of cancer risk. It might be a subject of future studies to establish whether zinc intake modifications changing Zn blood level will be an effective way of cancer prevention.

## A18 Copper as marker of cancer risk in *BRCA1*(+) women in Poland

### Muszyńska M^1,2^, Marciniak W ^1,2^, Derkacz R.^1,2^, Kuświk M ^2^, Baszuk P ^1,2^, Białkowska K ^2^, Huzarski T ^1,2^, Gronwald J^1,2^, Oszurek O ^2^, Cybulski C ^1,2^, Dębniak T ^2^, Tołoczko-Grabarek A^1,2^, Morawski A ^1^, Jakubowska A^2,3^, Lubiński J. ^1,2^

#### ^1^READ-GENE S.A., Grzepnica, Poland; ^2^Department of Genetics and Pathology, International Hereditary Cancer Center, Pomeranian Medical University, Szczecin, Poland; ^3^Independent Laboratory of Molecular Biology and Genetic Diagnostics, Pomeranian Medical University, Szczecin, Poland

Introduction

Copper is a micronutrient necessary for functioning of the organisms. Copper homeostasis is tightly regulated and is hard to change. The higher copper level are observed in different conditions, including inflammation and cancer. In literature there are 7 studies concerning Cu level and cancer in prospective study, the latest from 2006. Those papers show inconlusive data [1-7].The study was conducted to determine if blood copper level could be a risk factor in developing cancer.

Material and methods

In our study, in a cohort of 648 women, *BRCA1* mutation carriers, during the average period of 48 months of observation 85 new cancers of different location were developed.Copper was quantitatively measured in diluted blood samples by inductively coupled plasma mass spectrometry (ICP-MS) using mass spectrometer (Elan DRC-e, PerkinElmer) in DRC mode.

Results

In the whole studied group statistically significant almost 2-fold increased risk of developing cancer for higher than 865 μg/l of copper concentration in blood was observed (OR=1,72; p=0,0268; 95% CI:1,081 – 2,759). The risk was more than 3-fold increased for above Cu concentration in blood for *BRCA1* mutation carriers in the age under 50 years (OR=3,28; p=0,0002; 95% CI=1,698 – 6,32)

Conclusions

The high copper level is a good marker of increased cancer risk for BRCA(+) women under 50 years of age. Further investigations are needed if decreasing blood copper level will be beneficial for those women.

References

1. Coates R.J., et al.: Cancer risk in relation to serum copper levels. *Cancer Res.* (1989); 49, s. 4353 – 4356.
2. Garland M., et al.: Toenail trace element levels and breast cancer: a prospective study. *Am. J. Epidemiol.* (1996); 144(7): 653 – 660.
3. Haines A.P., et al.: Cancer, retinol binding protein, zinc and copper. *Lancet* (1982); 1(8262): 52 – 53.
4. Kok F.J., et al.: Serum copper and zinc and the risk of death from cancer and cardiovascular disease. *Am. J. Epidemiol.* (1988); 128(2): 352 – 359.
5. Leone N., et al.: Zinc, copper, and magnesium and risk for all-cause, cancer, and cardiovascular mortality. *Epidemiology* (2006); 17(3): 308 – 314.
6. Overvad K., et al.: Copper in human mammary carcinogenesis: a case-cohort study. *Am. J. Epidemiol.* (1993); 137(4): 409 – 414.
7. Wu T., et al.: Serum iron, copper and zinc concentrations and risk of cancer mortality in US adults. *Ann. Epidemiol.* (2004); 14(3): 195 – 201.

## A19 Cadmium as a marker of cancer risk

### Marciniak W, Muszyńska M^1,2^, Derkacz R^1,2^, Kuświk M ^2^, Baszuk P^1,2^, Białkowska K^2^, Huzarski T^1,2^, Gronwald J^1,2^, Oszurek O^2^, Cybulski C^1,2^, Dębniak T^2^, Tołoczko-Grabarek A^1,2^, Morawski A^1^, Jakubowska A^2,3^, Lubiński J^1,2^

#### ^1^READ-GENE S.A., Grzepnica, Poland; ^2^Department of Genetics and Pathology, International Hereditary Cancer Center, Pomeranian Medical University, Szczecin, Poland; ^3^Independent Laboratory of Molecular Biology and Genetic Diagnostics, Pomeranian Medical University, Szczecin, Poland

Material and Methods

Three prospective cohorts of A) BRCA1 carriers, B) women without detected pathogenic variants in genes involved in DNA repair pathway and C) men were set. Cohorts includes 601,1698,1467 initially unaffected patients, respectively. After a mean 48,54,20 months of follow-up the 42,110,103 cancers were observed in each cohort, respectively.

Samples were measured with an ICP-MS technique, using direct alkali dilution. Matrix matched calibration and internal standard were used.Conditional logistic regression and Chi-square test were used for end point assessment.

Results

In the BRCA1 carriers group we found significantly lowered risk for the lowest quartile of cadmium concentration (OR=0,2944; p=0,0346; 95%CI:0,08789-0,9858). Further analysis of collected data shows that this effect is increased if cadmium level is below 0,32 μg/l and leads to 9 times lowered risk of cancer if women are below 51 yr (OR=0,1136; p=0,0075; 95%CI: 0,0151-0,8553).

In the group of women free of any pathogenic variants in DNA repair pathway we showed that higher levels of cadmium in blood might be beneficial for prospective risk of breast cancer risk. Analysis of our biggest cohort revealed that cadmium levels higher than 0,59 μg/l are associated with up to 4 times lower risk of breast cancer (OR= 4,20; p=0,0319; 95%CI:1,01-17,80).

Prospective observation of men reveals that lowest quartile of cadmium concentration compared to the sum of the rest quartiles gives us 3 times lower risk for any cancer (OR=0,2974; p=0,0009; 95%CI:0,1424-7,025)

Conclusions

There is a pending need for cadmium removal therapy in women carrying BRCA1 founder mutation and men. Result from cohort of healthy women without genetic mutations of susceptibility for breast cancer is in opposite to current state of knowledge but it is confirmed in two independent studies. Further research on evaluating breast cancer risk in women without BRCA1 mutation is needed.

## A20 Arsenic as a marker of cancer risk

### Derkacz R^1,2^, Marciniak W^1,2^, Kuświk M^2^, Baszuk P^1,2^, Muszyńska M^1,2^, Białkowska K^2^, Lener M^2^, Huzarski T^1,2^, Cybulski C^1,2^, Gronwald J^1,2^, Morawski A^1^, Oszurek O^2^, Tołoczko-Grabarek A^1,2^, Dębniak T^1,2^, Jakubowska A^2,3^, Lubiński J^1,2^

#### ^1^Read-Gene S.A., Grzepnica, Poland; ^2^Department of Genetics and Pathology, International Hereditary Cancer Center, Pomeranian Medical University, Szczecin, Poland; ^3^Independent Laboratory of Molecular Biology and Genetic Diagnostics, Pomeranian Medical University, Szczecin, Poland

Introduction

Trace amounts of arsenic are considered as necessary for normal development of organism. Animals study shows inverse correlation between arsenic and toxic effects. Exclusion of this element from food causes in animals among others depressed growth and abnormal reproduction [1,2]. Arsenic is generally considered as a cancer risk factor (kidney [3], bladder [4], skin [5] and lung cancer [4]).

Methods

Four prospective groups were analyzed. The cohort of women BRCA1 carriers consists of almost 601 initially unaffected with 42 cases developed during the 4 years of follow-up. The cohort of women BRCA1 non-carriers consists of almost 1698 initially unaffected with 110 cases developed during 5 years of follow-up. The men cohort consists of almost 1467 initially unaffected with 103 cases developed the 2 years of follow-up.

Blood arsenic level was measured by ICP-MS Elan DRC-e (PerkinElmer).

Results

Low arsenic blood level (AB) in prospective cohort of women *BRCA1* carriers and women *BRCA1* non-carriers decrease risk of cancer respectively 4 (AB < 0.65 μg/L, p=0.004, OR=4.10, 95%CI: 1.1-19.15) and over 29 (AB < 0.59 μg/L, p=0.0001; OR=29.50, 95%CI: 4.10-212.00) times.

The results of men prospective cohort are unexpected. Among men with arsenic blood level exceeding 1.40 μg/L, risk of cancer is decreased more than 5 times (p=0.0482, OR=5.768, 95%CI: 0.7821-42.539).

Conclusions

Results from this study suggests that arsenic blood level can be the strongest diet marker of cancer risk among women and men in Poland. Diet modifications changing blood arsenic level may be effective way of cancer prevention.

References

[1] Molin M, Ulven SM, Meltzer HM, Alexander J, Arsenic in the human food chain, biotransformation and toxicology – Review focusing on seafood arsenic. J Trace Elem Med. Biol 2015;31:249-59

[2] Nielsen FH, Importance of making dietary recommendations for elements designated as nutritionally beneficial, Pharmacologically Beneficial, or Conditionally Essential, J Trace Elem Med. Biol 2000;13:113-129

[3] Hopenhayn-Rich C., Biggs M.L., Fuchs A., Begoglio R., Tello E.E., Nicolli H., Smith A.H.: Bladder cancer mortality associated with arsenic in drinking water in argentina. *Epidemiology* (1996); 7(2), s. 117 – 124.

[4] Mostafa M.G., McDOnald J.C., Cherry N.M.: Lung cancer and exposure to arsenic in rural Bangladesh. *Occup. Environ. Med.* (2008); 65(11), s. 765 - 768.

[5] Karagas M.R., Stukel T.A., Morris J.S., Tosteson T.D., Wei J.E., Spencer S.K., Greenberg E.R.: Skin cancer risk in relation to toenail arsenic concetration in a US population-based case-control study. *Am. J. Epidemiol.* (2001); 153(6), s. 559 – 565.

## A21 SELINA – clinical trial on lowering the risk of malignancies by optimizing selenium levels in females from families with hereditary breast cancer*

### Lubinski J^1,2^

#### ^1^Department of Genetics and Pathology, Pomeranian Medical University in Szczecin, Szczecin, Poland; ^2^Read-Gene SA, Grzepnica, Poland

Aim

Prospective observational studies showed that blood selenium (Se) levels associated with significantly lower risk of cancers can be identified in Polish females from families with hereditary breast cancers (HBC). For BRCA1 mutation carriers it is: 70-89 μg/l at age <50 yrs (OR~12) and 95-120 μg/l at age ≥50 yrs (OR~4). For females without detected BRCA1 mutation but from families with pedigree/clinical features of HBC it is 98-108 μg/l (OR~5).

The main goal of SELINA is validation of hypothesis that optimization of Se level by supplementation or diet changes can decrease the risk of malignancies in groups described above.

Method

7000 females (including 1200 BRCA1 carriers) from families with HBC and deficiency or excess of Se will be recruited and randomly qualified to one of the following arms: “placebo”, prospective observational, supplement (Sodium Selenite) or diet modification. Blood Se level will be systematically measured using ICP-MS and appropriately optimized. Follow-up will take 5 yrs.

Results

At present we are performing recruitment. It is planned to close it at the end of 2018.

Conclusion

SELINA is the first clinical trial aimed to decrease the risk of cancers by active control of blood selenium levels. All interested scientists/institutions are welcome for collaboration.

*Project - INNOMED/I/16NCBR/2014 sponsored by National Ctr of Research & Development and Read-Gene SA.

## A22 Rules of medical management for patients with BRCA1 gene mutation

### Gronwald J^1^

#### ^1^Department of Genetics and Pathology, Pomeranian Medical University in Szczecin, Szczecin, Poland

Molecular diagnostics of patients with familial aggregation of breast and ovarian cancers have more than 20 years of history. At that time in Department of Genetics and Pathology of the Pomeranian Medical University in Szczecin we diagnosed around 8,000 patients with the BRCA1 and BRCA2 gene mutation and analyzed factors affecting the risk of cancer as well as assessed optimal methods for early detection of cancer and their effective treatment. Based on our own observations and other researchers, we discuss the current principles of prophylaxis, cancer detection and treatment in carriers of the BRCA1 and BRCA2 gene mutations.

## A23 Evaluation of the constructed device along with the software for digital archiving, sending the data and supporting the diagnosis of cervical cancer

### Gronwald J^1,2^_,_ Barbasz J^1,3^, Lasyk Ł^1^, Żuk P^1,4^, Prusaczyk A^1,4^, Włodarczyk T^1, 4^, Prokurat E^1,4^, Olszewski W^5^, Bidziński M^6^

#### ^1^Digitmed Sp. z o.o., Oleśnica, Poland; ^2^Department of Genetics and Pathology, Pomeranian Medical University in Szczecin, Szczecin, Poland; ^3^Institute of Catalysis and Surface Chemistry Polish Academy of Sciences, Cracow, Poland; ^4^Centrum Medyczno-Diagnostyczne Sp. z o.o., Siedlce, Poland; ^5^Department of Pathology, Maria Skłodowska-Curie Memorial Cancer Center and Institute of Oncology, Warsaw, Poland; ^6^Oncological Gynaecology Clinic, Maria Skłodowska-Curie Memorial Cancer Center and Institute of Oncology, Warsaw, Poland

The incidence and mortality of cervical cancer are high in Poland. There are effective methods of the prevention and the early diagnosis, however those methods require well-trained medical professionals including cytologists.

Within this project we designed and built a prototype of a new device together with implemented software to convert the currently used optical microscopes to fully independent scanning systems for cytological samples. The use of the device is intended to improve the effectiveness of cytological screening, and registration of cytological tests’ results. The features of the software include digital backup as well as transmission and telemedicine evaluation.

We proposed the use of a specific method to support diagnostics. The first goal was to decrease time of analysis in the entire process. The second goal was to achieve a quality of recognition of positive research results at least comparable with cytologists.

To evaluate the quality of the device we compared 8 000 conventional cervical cytology augmented by computer-assisted image analysis system vs. manually read as well as liquid based cytology augmented by computer-assisted image analysis system vs. manually read. The following and economic outcome measures were evaluated: clinical - detection rates, relative sensitivity, sensitivity difference, specificity difference; economic - cost per test, workload per test, acceptability of the computer-assisted image analysis system in the screening services, organizational and budget impact of computer-assisted image analysis system, as well as social and economic benefits (positive externalities).

This project is co-financed with European Regional Development Fund within the Priority Axis I, Support for R&D activities for companies, Measure 1.2, Sectoral R&D Programmes, Sectoral Programme: “INNOMED – scientific research and development programme for innovative medicine economy sector”.

Funding

Międzynarodowa Konferencja „Genetyka Kliniczna Nowotworów 2018”- zadanie finansowane w ramach umowy 1006/P-DUN/2018 ze środków Ministra Nauki i Szkolnictwa Wyższego przeznaczonych na działalność upowszechniająca naukę.

International Conference „Clinical Genetics of Cancer 2018” - task financed under the agreement 1006/P-DUN/2018 from the funds of the Minister of Science and Higher Education designated for dissemination activities.



